# Advancing personalized medicine for tuberculosis through the application of immune profiling

**DOI:** 10.3389/fcimb.2023.1108155

**Published:** 2023-02-10

**Authors:** Vo Thuy Anh Thu, Ly Da Dat, Rannissa Puspita Jayanti, Hoang Kim Tu Trinh, Tran Minh Hung, Yong-Soon Cho, Nguyen Phuoc Long, Jae-Gook Shin

**Affiliations:** ^1^ Department of Pharmacology and PharmacoGenomics Research Center, Inje University College of Medicine, Busan, Republic of Korea; ^2^ Center for Personalized Precision Medicine of Tuberculosis, Inje University College of Medicine, Busan, Republic of Korea; ^3^ Center for Molecular Biomedicine, University of Medicine and Pharmacy at Ho Chi Minh, Ho Chi Minh City, Vietnam; ^4^ Department of Clinical Pharmacology, Inje University Busan Paik Hospital, Busan, Republic of Korea

**Keywords:** tuberculosis, immunomics, multi-omics, personalized medicine, biomarkers, model-informed precision dosing

## Abstract

While early and precise diagnosis is the key to eliminating tuberculosis (TB), conventional methods using culture conversion or sputum smear microscopy have failed to meet demand. This is especially true in high-epidemic developing countries and during pandemic-associated social restrictions. Suboptimal biomarkers have restricted the improvement of TB management and eradication strategies. Therefore, the research and development of new affordable and accessible methods are required. Following the emergence of many high-throughput quantification TB studies, immunomics has the advantages of directly targeting responsive immune molecules and significantly simplifying workloads. In particular, immune profiling has been demonstrated to be a versatile tool that potentially unlocks many options for application in TB management. Herein, we review the current approaches for TB control with regard to the potentials and limitations of immunomics. Multiple directions are also proposed to hopefully unleash immunomics’ potential in TB research, not least in revealing representative immune biomarkers to correctly diagnose TB. The immune profiles of patients can be valuable covariates for model-informed precision dosing-based treatment monitoring, prediction of outcome, and the optimal dose prediction of anti-TB drugs.

## 1 Introduction

Accounting for approximately 1.4 million fatal cases in 2021, tuberculosis (TB) is the 13^th^ leading cause of death and the second most common life-threatening infection after coronavirus disease 2019 (COVID-19) (WHO, 2022). Knowing no place or age makes TB a significant threat to public health, especially in overpopulated or developing countries. Commonalities of 30 high-TB-burden countries and three global TB-watchlist countries (WHO, 2022) include the pressures of either overwhelming medical demand or financial instability. Therefore, a reliable, fast, and affordable medical approach to this curable and preventable disease is the key to controlling the TB epidemic.

The WHO’s End TB Strategy encompasses three pillars centralizing the patients, encouraging government-communities cooperation, and intensifying innovative research to eventually hit the goals of an 80% drop in TB incidence, a 90% drop in TB mortality, and catastrophic cost elimination for TB-affected households by 2030 in comparison to 2015. Reaching these milestones would have required a constant incidence decline of 4-5% per year by 2020 and a further drop of 10% by 2025 (WHO, 2022). However, the reality of current records fails to keep track of the End TB Strategy’s targets. Meanwhile, TB-related mortality reduction between 2015 and 2020 was only a quarter of the projected 2020 milestone of 32%. TB deaths increased in 2020, the first such occurrence for nine straight years. This resulted from the restriction of medical access during the COVID-19 pandemic. In particular, nearly half of TB cases were not diagnosed and treated (WHO, 2021b); undetected TB rose, which, with some lag-time, eventually caused an increase in TB death, community transmission, and the number of people developing TB (WHO, 2022). Hence, regarding the fact of our failure to reach the WHO’s target and the dominance of TB in developing countries, it is a must to optimize the existing tools and studies for the next generation to be predictable, fast, and affordable in giving access to all affected countries and populations, and helping to return to the expected trajectory of the global TB epidemic.

Culture conversion or sputum smear microscopy is still widely used regardless of their limitations for diagnosing and monitoring treatment responses in adults with pulmonary TB (PTB) (Yong et al., 2019; Zimmer et al., 2022). These methods require sputum samples, which are not promptly accessible from all populations, have long turnaround times, and are prone to contamination with poor specificity and sensitivity for outcome prediction (Yong et al., 2019; Zimmer et al., 2022). Furthermore, some patients face a risk of infection recurrence or have a higher risk of acquiring multi- or extensive drug-resistant TB (M/XDR-TB), especially those from low-income countries. Additionally, challenges regarding the accuracy of sputum-based diagnostics are often raised in cases of paucibacillary related to human immunodeficiency virus (HIV) and childhood TB (Newton et al., 2008; Cattamanchi et al., 2009). Hence, the WHO has endorsed several non-sputum-based TB diagnoses by utilizing the urine or blood of TB patients through nucleic acid amplification tests and loop-mediated isothermal amplification (LAMP) reaction, which have appeared as promising point-of-care (POC) tests for TB (Organization, 2020). Additionally, the tuberculin skin test (TST) and interferon-gamma release assays (IGRAs) have been widely utilized for latent tuberculosis infection (LTBI) detection in clinical practice (Zellweger et al., 2020), but neither of these effectively distinguish LTBI from active TB (Sharma et al., 2017). Even though IGRAs offer superior specificity, they are more expensive and associated with organizational and logistical challenges (Zellweger et al., 2020). Therefore, there is an urgent need to develop a novel, low-cost, non-sputum-based TB diagnostic test that is sensitive and specific, and employs readily available biological materials such as blood or urine (Goletti et al., 2016; Goletti et al., 2018).

The polymerase chain reaction (PCR)-based technique, namely GeneXpert MTB/RIF, recently developed to amplify the *Mtb* gene, represents a significant advancement in diagnosing TB (Zeka et al., 2011; Kwak et al., 2013) and detecting rifampicin resistance (Organization, 2020) within two hours. Sensitivity and specificity of PTB detection up to 79,5% and 100%, respectively, were reported by Kwak’s group when employing GeneXpert MTB/RIF assay in clinical practice (Kwak et al., 2013). Nevertheless, the utilization of the test limits the diagnosis of active PTB, not LTBI. Moreover, both PCR-based and sputum-based diagnostic systems exhibit inherent limitations in that they rarely detect extrapulmonary (EP)-TB in an efficient manner (Lee and Diseases, 2015). Therefore, an approach focused on the host biomarkers involved in host immune responses and pathological processes of LTBI, active TB, and EP-TB could be a better method for diagnosis and treatment monitoring.

**Figure 1:**
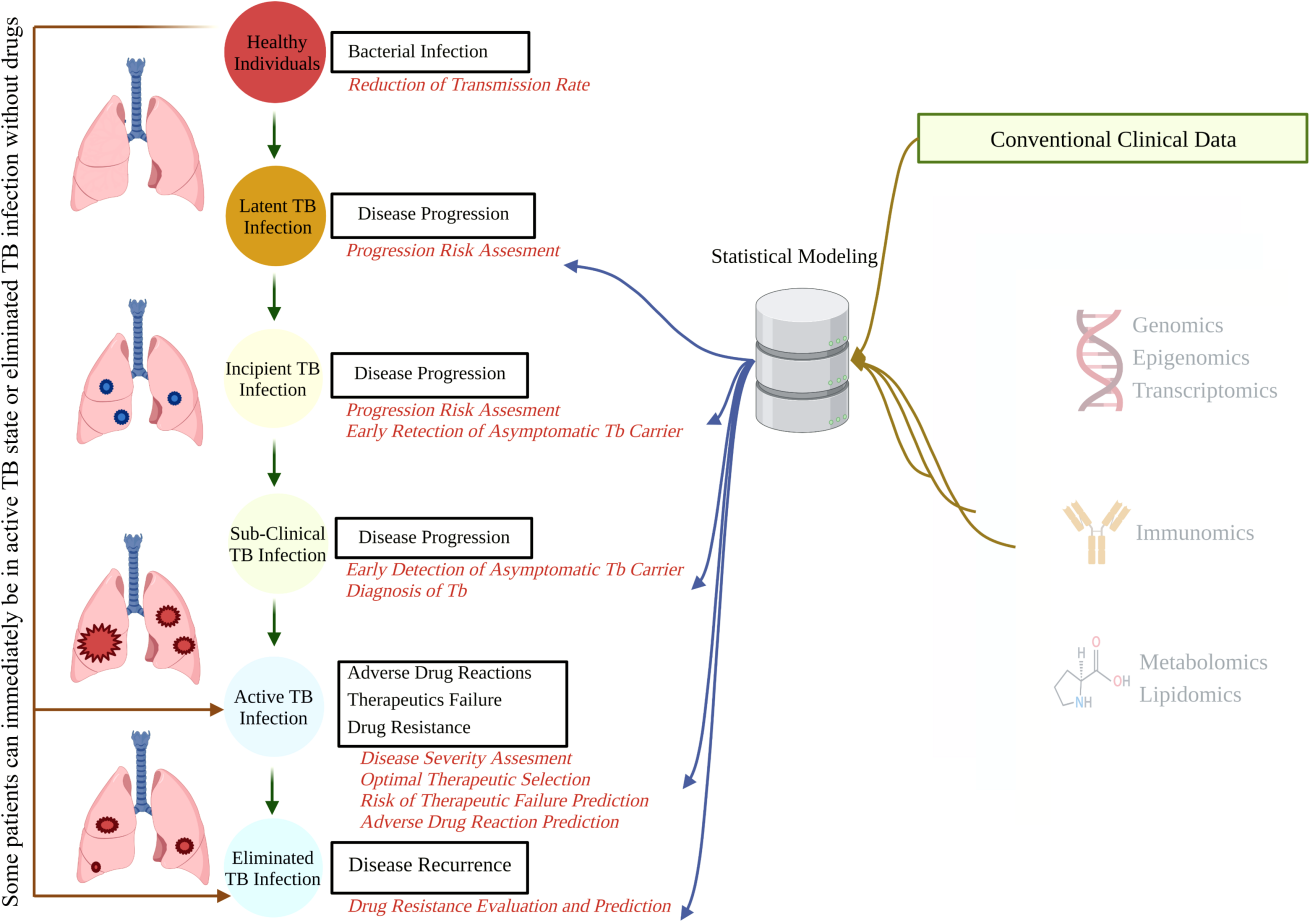
The natural course of TB and potential points for biomarker-guided interventions. A better understanding of disease progression, accruable diagnosis, appropriate monitoring, and possible therapeutic interventions can be acquired by integrating demographic and conventional clinical data with high-throughput omics data. TB, tuberculosis.

As a predictable ending readout for various biological studies such as genomics, transcriptomics, proteomics, and metabolomics, omics sciences aim to quantify and demonstrate the biological profiles related to the structure, function, status, and dynamics of a cell, tissue, or organism (Vailati-Riboni et al., 2017). Immunomics is the study of immune responses after the interface of pathogen-derived proteins and the host immune system, focusing on multiple aspects of the immune molecules, such as their presence, variation, target, and function at a certain stage of a health condition (Doolan et al., 2014; De Sousa and Doolan, 2016; Yan et al., 2018). Immune profiling has provided valuable complementary information for genomics, transcriptomics, and proteomics (**Figure 1**). This advanced combination has provided an unprecedented opportunity to develop a rational immunomic approach to target antigen selections such as *Plasmodium* (Doolan, 2011; ENPICOM, 2020), COVID-19, and TB, which are all significant public health problems and pose a threat to complex genetic and immunological adaptations (De Sousa and Doolan, 2016; Yan et al., 2018).

TB has remained in the community (Barberis et al., 2017) due to the lack of highly effective biomarkers of the host which has restricted the improvement of diagnoses and treatment strategies. Several immune responses have been reported as a part of natural defense mechanisms against TB, such as interferon-γ (IFN-γ) production, CD4^+^ T cell or polyfunctional T cell responses, interactive immunogenic secretion, and regulation. Therefore, the evaluation of host immune responses by immune profiling, including cytokines, chemokines, the proteins associated with responses to inflammation or proteins released during TB evasion, tissue damage, and treatment prognosis, will shed light on TB diagnosis and the clinical management of individual patients (Walzl et al., 2011).

Moreover, besides being a supportive information channel, immune response profiling claims certain advantages compared to current gene-expression profiling and its precedent counterparts such as ELISA (Enzyme-Linked Immunosorbent Assay). By directly targeting expressive proteins, which are immune molecules, the approach bypasses the concern of relatively low transcriptional correlation (less than 0.3 among 1,066 genes in 23 human cell lines) (Gry et al., 2009) in genomic and transcriptomic studies. The unavoidable presence of fluctuated metabolites during translation and post-modification is another setback that immunomics scientists can generally ignore. Finally, by using advanced technology, high-throughput immune profiling requires much lower blood volume than DNA isolation does in genomic assay or ELISA (approximately 50 μL and hundreds μL, respectively) (Elshal and McCoy, 2006). It also shortens the running time significantly from days to hours, the turnaround time for laboratory diagnosis, and reduces the number of running plates several times compared to ELISA, all of which end up reducing the workload for blood collection and other clinical practices. For those reasons, high-throughput immune profiling can offer enormous potential as either a sufficient reference for TB management or an additional input for better and more precise patient stratification.

In this article, we summarize the current discovered immune markers and immune-centric approaches for TB investigation in the omics era. Furthermore, we propose a novel TB-management strategy through semi-automated therapeutic drug monitoring (TDM) utilizing model-informed precision dosing (MIPD).

## 2 Prospects for TB-associated biomarkers investigations

In clinical care for TB, the demand for biomarkers is most critical for certain investigations, including the prediction of durable cure in active disease cases, disclosing reactivation risk, monitoring treatment, predicting outcome for latent *Mtb-*infected individuals, and vaccine efficacy prediction in case of patients suffering from other active diseases (Wallis et al., 2010). Furthermore, biomarkers can benefit candidate determination during drug discovery, accelerating dosage selection in preliminary clinical research and enriching the knowledge of TB pathogenesis (Wallis et al., 2010).

### 2.1 Host humoral responses to *Mtb* infection

Infection and the host immune system have an intimate relationship as two facets of the same coin. When *Mtb* enters the host lung *via* inhalation and invades interstitial tissue, the immune response is inevitably activated, resulting in host profile alterations (Bulterys et al., 2019; Yong et al., 2019). Since they are easy to use, affordable, and practical for POC, pathogen-specific antibodies are most often utilized as the host biomarkers for serologic tests. The *Mtb*-specific antibodies have been extensively investigated for infection diagnosis and response prediction to anti-TB treatment, including IgA, IgG, and IgM antibodies to: mycobacterial secreted 38-kDa and cytosolic 16-kDa antigens (Raja et al., 2008; Ben-Selma et al., 2011); antibodies against culture filtrate protein 10 (CFP-10) and early secretory antigenic target 6 (ESAT-6) (Andersen et al., 2000; Arend et al., 2000); and antigen 60, heat shock protein, purified protein derivative, and lipid-derived antigens (Kumar Verma and Jain, 2007). Even though participants had smear-positive results, earlier investigations revealed a low sensitivity diagnosis, approximately 75% at best, by ELISA (Raja et al., 2008). The heterogeneous quality and quantity of host antibody responses could be revealed as the main reason, and probably arise at different stages of the disease or are regulated by multiple host factors. Recently, several promising candidates were reported to improve the sensitivity and specificity of TB diagnosing assays, some widely common, such as: serum IgG against lipoarabinomannan (LAM) (Ben Selma et al., 2010); RV1255c-E and RV0310c-E antigens (Luo et al., 2017); proline-proline-glutamic acid protein 17 (Abraham et al., 2018); and mycobacterial DNA binding protein 1 (Maekura et al., 2019).

### 2.2 Host cellular immune responses to *Mtb* infection

Host immunity protection against *Mtb* is not fully understood, but it depends on various innate and adaptive immunological mechanisms. Additionally, cellular immune responses to *Mtb*-specific antigens have demonstrated higher constancy than antibody responses (Yong et al., 2019). In principle, T cell-induced immune responses play a crucial role in controlling and protecting against *Mtb* infection (Walzl et al., 2011). At certain differentiated stages, CD4^+^ and CD8^+^ T cells progress to central memory (T_CM_) and effector memory (T_EM_), which activate and modulate phagocytosis and produce molecules that can directly influence cytotoxic microbial infection. Based on these understandings, several attempts have been undertaken to characterize the T cell-functional signature correlated with different TB stages. In particular, CD69 or CD137, a co-stimulatory receptor, is responsible for T cell activation and proliferation, and the frequency of CD69^+^ and CD137^+^ has been reported to link with active TB (Nikolova et al., 2013; Yan et al., 2017). Another study by Ahmed et al. used two markers for immune activation, CD38, and maturation, CD27, on *Mtb*-specific CD4^+^ T cells to differentiate active TB and LTBI (Ahmed et al., 2018). According to their observations, CD38^pos^ CD27^low^ frequencies were increased in active TB whilst LTBI performed CD38^neg^ CD27^high^ profile.

Treatment efficacy can be monitored *via* promising surface markers such as T-cell activation (CD38, HLA-DR) and proliferation (Ki-67) (Adekambi et al., 2015) under phenotypic characteristic analysis on *Mtb*-specific T cells. After nine weeks of anti-TB therapy initiation, CD38, Ki67, and HLA-DR frequencies of T cells were remarkably decreased and associated with stable culture conversion time (Ahmed et al., 2018). The amount of T_EM_ and T_CM_ could also be exerted as a predictor for reoccurrence, in which after six months of anti-TB therapy initiation the cases with a substantial amount of T_EM_ may result in relapse because of living *Mtb* persistence, while T_CM_ may reflect the complete clearance of *Mtb* (Millington et al., 2010; Wang et al., 2010).

### 2.3 Host cytokine and chemokine responses to *Mtb* infection

Various host cytokines and chemokines are released once *Mtb*-specific antigens encounter and stimulate the immune responses of an *Mtb*-infected individual (Baatjies et al., 2021). The intricate relationship between *Mtb* and the immune system is attributed to the production of various cytokines in response to *Mtb* infection (Etna et al., 2014) which could restrict bacterial growth and modulate inflammatory responses that contribute to the development of TB (Bai et al., 2018). Additionally, chemokines are supposed to be closely associated with the development and maintenance of quiescent granulomas as well as enrolling periphery cells to positioning within the granuloma (Kumar et al., 2019b). Diverse investigations have focused on cytokine and chemokine identification which can improve discrimination between LTBI and active TB, treatment monitoring, and outcome prediction (**Table 1**).

**Table 1:** Representative reported immune biomarkers in laboratory and clinical scopes.

Purpose	Potential biomarkers	Target functions	Ref
Diagnosis	IL-2, IP-10, IL-5, IL-13, IFN-γ, IL-10, TNF-α	Active TB and LTBI	(Wei et al., 2020)
	TNF-α, IL-6, IP-10, IFN-γ, and MIP-1β	PTB, EB-TB, TP, and HC	(Xiong et al., 2016)
	CCL1, CCL3, CXCL1, CXCL10	PTB, LTBI, and HC	(Kumar et al., 2019b)
	IL-6, IP-10, TNF-α, sCD163, and sCD14.	Active TB, LTBI, HC	(Zambuzi et al., 2016)
	Eotaxin, MIP-1α, MDC, IP-10, MCP-1, IL-1α, IL-10, and TNF-α	Active TB and LTBI	(Luo et al., 2019)
	IL-4, IL-5, IL-10, IL-13, and IL-37	PTB, LTBI, and HC	(Moideen et al., 2020)
	IFN-γ, TNF-α, IL-17A, and IL-1β	PTB, LTBI and HC	(Kumar et al., 2019a)
	IL-1, IL-6, IL-10, IL-12, IL-4, IL-2, IL-15, IFN-γ, MMP-1, MMP-9	PTB and HC	(Liu et al., 2018)
	IL-3, IL-12-p40, LIF, IFN-α-2, IL-2Rα, β-NGF, SCF, TNF-β, TRAIL, IL-2, IFN-γ, IP-10, MIG, MIF, IL-13	active TB, LTBI, and non-TB patients	(La Manna et al., 2018)
	IL-8, IP-10, MIP-1α, sIL-2Rα, VEGF, MCP-3; G-CSF, GM-CSF, IL-1α, IL-2, BCA-1, and Eotaxin-1	Active TB and LTBI and HC	(Yao et al., 2017)
DR-TB	CRP, SAA, VEGF, IL-2Rα, IP-10 (CXCL10), MCP-1/CCL2	MDR-TB	(Ferrian et al., 2017)
	IFN-γ, TNF-α, and IL-10	DR-TB and DS-TB	(Basingnaa et al., 2018)
Treatment monitoring	CRP, IP-10, IL-6, and TNF-α	TB treatment response during 8 weeks	(Zimmer et al., 2022)
	combination of MCP-1/CCL2, IP-10, sIL-2Rα, SAA, CRP	Monitoring for MDR-TB (Month 0, 2, 4, 6)	(Ferrian et al., 2017)
	IL-6, CRP, IFN-γ, and TGF-β	Mornitoring treatment (Month 0, 2, 4, 6, 9)	(Díaz et al., 2017)
	SAA1, PCT, IL-1β, IL-6, CRP, PTX-3, and MMP-8	Monitoring treatment (week-8, week-12)	(Sigal et al., 2017)
	IP-10, MCP-3, IL-6, IL-1RAs, IL-1α,	Monitoring treatment (week-8, week-16)	(Rambaran et al., 2021)
	IP-10, and RANTES (CCL5)	PTB and HC, monitoring (0.5, 2, 4, and 6 months treatment)	(Zhao et al., 2018)
Outcome prediction	IL-6, IP-10, sCD14, and IFN-γ	TB recurrence and non-TB recurrence	(Sivro et al., 2017)

DR-TB, drug resistant tuberculosis; DS-TB, drug susceptible tuberculosis; EB-TB, endobronchial tuberculosis; HC, healthy control; LTBI, latent tuberculosis infection; MDR-TB, multi drug resistant tuberculosis; PTB, pulmonary tuberculosis; TB, tuberculosis; TP, tuberculous pleurisy.

The most popular commercial immune-based diagnosis for TB in clinical practice is still IGRAs despite the fact that IGRAs do not discriminate between active TB and LTBI and are perhaps inefficient in high-burden conditions (Organization, 2011; Pai et al., 2014; Jacobs et al., 2016). Hence, numerous studies have been reported to identify several singleplex or complex accurate and sensitive cytokine-based biomarkers for serological tests. In particular, cytokine/chemokine-based biomarkers can be used for monitoring treatment response in TB patients. Another examination was performed with plasma chemokines of PTB, LTBI, and healthy control (HC) individuals (Kumar et al., 2019b) to determine mycobacterial burden and disease severity. This study exhibited that the PTB chemokine levels containing CCL1, CCL3, CXCL1, CXCL2, CXCL9, and CXCL10 are significantly higher compared to LTBI and HC individuals. Furthermore, Kumar et al. also found that the plasma levels of CCL1, CCL2, CXCL2, CXCL9, CXCL10, and CXCL11 are all remarkably reduced following anti-TB chemotherapy, suggesting that they can be added to host chemokine panels for treatment-response monitoring. A meta-analysis was performed to identify valuable cytokines for distinguishing LTBI and active TB (Wei et al., 2020). Fourteen studies with 982 participants evaluated AUC, sensitivity, and specificity to reveal potential immune markers. As a result, seven molecules were identified that can discriminate between individuals with LTBI and active TB, including IL-2 (AUC, 0.91; sensitivity, 0.87; specificity, 0.61), IP-10 (0.86, 0.77, and 0.73), IL-5 (0.85, 0.64, and 0.75), IL-13 (0.85, 0.75, and 0.71), IFN-γ (0.80, 0.67, and 0.75), IL-10 (0.80, 0.68, and 0.74), and TNF-α (0.78, 0.67, and 0.64). Notably, due to their low specificity, single cytokines barely exhibit adequate diagnostic performance to be used as biomarkers. Also, host immune signatures have been developed in primary care settings (Mutavhatsindi et al., 2021). However, there is insufficient evidence to support their validity in secondary care settings.

Primarily evidence revealed that the host immune response to *Mtb* antigens is suppressed in resistant TB, but inflammatory cytokines still serve a fundamental role in the persistence of pulmonary tissue damage (Fortes et al., 2005). Impaired Th1-related cytokine production (Lee et al., 2002; Fortes et al., 2005; Geffner et al., 2009), such as IFN-γ, and increased Th2-related cytokines (Geffner et al., 2009), such as IL-4, were observed early in blood samples collected from MDR- and drug susceptible TB (DS-TB) patients, which emphasizes the alteration of Th1/Th2 profile characteristics in the disease progression. Furthermore, at the initiation of treatment for MDR-TB patients, peripheral Th1 and Th2 cells are remarkably diminished in comparison with drug susceptible TB (DS-TB) and HCs. These observations suggested the inhibition of both Th1 and Th2 pathways were intervened in the host response to MDR-TB infection (Tan et al., 2012). Besides the decreased level of IFN-γ, it was also found that IL-2 cytokines are significantly diminished in MDR-TB blood, even though they can function as protective cytokines against MDR-TB infection. Notably, different cytokine profiles are characterized in each stage of MDR-TB patients, with significantly decreased levels of IFN-γ, IL-2, and IL-10 in early infection, while during progressive necrosis and fibrosis, IL-4, IL-6, and TNF-α are increased (Tan et al., 2012). These results show that MDR-TB infection is undoubtedly complicated and challenging to treat. Thus, it is necessary to undertake extensive examinations for MDR-TB cytokine profiles that can identify biomarkers for MDR-TB diagnosis and treatment monitoring.

There are fluctuating cytokine profiles that coincide during the treatment process from the transition between intensive TB treatment and the continuation phase (Riou et al., 2012). The data from the study of Riou and colleagues demonstrated that only plasma levels of IP-10 and vascular endothelial growth factor (VEGF) remarkably changed in response to anti-TB treatment (Riou et al., 2012). These results substantiated previous observations that the reduction of plasma IP-10 is associated with successful TB treatment (Azzurri et al., 2005; Djoba Siawaya et al., 2009); hence IP-10 could be utilized as a valuable indicator of clinical response and treatment success. Furthermore, an investigation was recently conducted that quantified serum inflammatory and anti-inflammatory cytokine concentrations and compared MDR-TB, DS-TB, and HC (Mensah et al., 2021). The results identified a low concentration of serum IFN-γ and a high level of IL-4 related to MDR-TB compared to DS-TB individuals. Moreover, in the measurement of proinflammatory and anti-inflammatory cytokines, Granzyme B, IFN-γ, IL-4, IL-6, and TNF-α showed the greatest promise to differentiate between DS-TB or MDR-TB and HC. A recent systemic review identified promising biomarkers for the effective assessment of TB therapy, indicating that TNF-α is the most valuable biomarker (Clifford et al., 2015). Moreover, the proportion of both IFN-γ and IL-2 was reported to increase during the treatment period, suggesting they may be common markers of immunological protection against TB. However, the cytokine profiles from reported studies that focused on treatment monitoring in TB are controversial due to the heterogeneity of study designs and small sample sizes (Clifford et al., 2015), so extensive investigation is required to predict any failure of first-line treatments and ensure the adequacy of LTBI.

Despite treatments initially seeming to be effective, TB relapse occasionally occurs after completing anti-TB therapy and clinical symptoms re-emerge (De Steenwinkel et al., 2013). Transition to a negative sputum culture is generally considered predictive for effective TB treatment after two months (Wallis et al., 2013). However, this predictive method has poor sensitivity, with only 40% predicting relapse and 57% predicting failure outcome (Horne et al., 2010). Recently, emerging studies indicated that the failures of the host immune system to restrain invading *Mtb*, including immune disorders or severe immunodeficiency, are supposed to be the leading cause of re-treated TB (Bai et al., 2018). Bai et al. observed that cytokines released from Th1 and Th2 cells dramatically altered and upregulated Th1/Th2 cytokine ratios in re-treated TB patients (Bai et al., 2018). Several serum markers, including IFN-γ, IL-2, IL-7, and soluble CD54, were shown remarkably enhanced and more sensitive compared with other cytokines. Hence, they might serve as helpful serum indicators to determine TB re-treatment response. Moreover, a study from Steenwinkel et al. compared plasma cytokine profiles of primary TB and re-occurrence TB, especially in patients who were treatment non-compliant (De Steenwinkel et al., 2013). Not only the *Mtb* load in infected organs, additionally, in the cases of HIV-infected individuals or patients suffering a long period of immunosuppressive therapy with TNF-α inhibitors, the reactivated risk of LTBI increased remarkably (Bruns et al., 2009; Getahun et al., 2010; Amelio et al., 2019). Hence, besides biomarkers that imply successful treatment during the therapeutic period, more specific indicators are required to predict relapse at the end of six-month treatment, which might significantly enhance clinical prognosis. Interestingly, an investigation from Ronacher et al. disclosed that the levels of IL-5 and MMP-2 decreased, while sIL-2Rα and CRP were highly elevated at diagnosis in relapse patients in comparison with failed and cured individuals (Ronacher et al., 2019).

## 3 Integration of immune profiling to the omics-based TB translational biomarker research

A multi-omics approach has facilitated the identification of biomarkers and provided a profound understanding of the pathological processes in the field of TB (Jakhar et al., 2020; Pitaloka et al., 2022). A multi-omics approach can provide a holistic view of disease mechanisms and better predict disease outcomes (Hasin et al., 2017; Ntoumi et al., 2022). Therefore, multi-omics has been recognized as one of the crucial pillars for personalized medicine for TB. The integration of genomics, transcriptomics, proteomics, and metabolomics approaches to monitoring TB treatment has been suggested recently (Pitaloka et al., 2022). However, as TB is an infectious disease that triggers the inflammation agents during the disease phases, immunomics plays a key role in the multi-omics approach (Bonaguro et al., 2022). Transcriptomic, metabolic, epigenetic, and functional immune studies should be integrated and further analyzed with correspondence to clinical outcomes data (Lange et al., 2020; Ntoumi et al., 2022). Although the biomarkers found have not been further validated in a larger cohort, so far the identified biomarkers hold promise for treatment monitoring, disease prediction, and differential diagnosis of TB (Yong et al., 2019; Perumal et al., 2021; Pitaloka et al., 2022).

### 3.1 TB management benefits from the utilization of immune biomarker panels

An immunomics approach has been widely investigated to differentiate between active TB, LTBI, and/or healthy people (Wei et al., 2020; Mutavhatsindi et al., 2021). Earlier publications found higher proportions of neutrophils and monocytes in active TB compared to non-active TB patients (Wang et al., 2015; La Manna et al., 2017; Estévez et al., 2020a). On the other hand, the proportions of T-helper (CD4^+^) cells and B cells were higher in non-active TB, suggesting that adaptive immune response is lower in active TB patients. However, as it appears more challenging, the immune expression profiles of those with LTBI largely overlapped with HC, making it difficult to distinguish separate clusters based on the 1,000 most variable genes (Bah et al., 2018). It was discovered that the type I and type II interferon signaling pathways’ upstream and downstream components are expressed more strongly in peripheral blood leukocytes from active TB patients (Berry et al., 2010; Zambuzi et al., 2016). Additionally, another study has shown that the expression of CD64, LTF, and Rab33A together formed biomarkers that can distinguish individuals with and without TB (Jacobsen et al., 2007). Furthermore, it has been widely recognized that IL-6 could be useful for monitoring the effectiveness of anti-TB drugs and, together with sCD14, used to distinguish the active state of the disease (Yong et al., 2019; Boni et al., 2022). Previous reports also showed that in active TB patients, plasma levels of sCD163 and TNF-α were linked with the severity of the condition and extent of lung damage (Zambuzi et al., 2016; De Andrade Júnior et al., 2008; De Steenwinkel et al., 2013). Another study also identified several increased immune markers were linked to active TB patients, reported as GM-CSF, IFN-α-2, IL-4, IL-5, IP-10, and MIP-1α (Zambuzi et al., 2016; Estévez et al., 2020b). Until now, the diagnosis confirmation of LTBI patients still followed the results of the IGRA test (Herrera et al., 2011). Nevertheless, several limitations, such as the large amount of blood needed, limited assay reproducibility, low sensitivity, unknown prognostic value, and slow sample processing, have limited IGRA usage for mass testing (Herrera et al., 2011; Kaul et al., 2022). A recent publication confirmed *FCGR1B, GBP1*, and *GBP5* as the transcript signatures that distinguished between LTB1 and active TB, which could be helpful in screening LTBI on a mass scale (Kaul et al., 2022). Furthermore, they classified the LTBI into clusters following the IFN-γ values in TB1 and TB2 antigens using machine-learning cluster analysis. Such a clustering approach may help to identify which group would likely progress to become active TB, thus effectively targeting preventive therapy in this cluster. However, the implementation of immunomics to distinguish between active TB and LTBI remains challenging. Even though these biomarkers could be further used to monitor treatment, differentially diagnose, and predict disease progression, little to none of these markers have been validated in extensive prospective studies. The ratio among biomarkers might keep changing inconstantly throughout the treatment phase and be considered as a dynamic of biomarkers, another critical challenge to be addressed (Dheda et al., 2010). Therefore, utilization of homologous (e.g., gene experiment only) or heterogenous (e.g., cytokines and endogenous metabolites) biosignatures to distinguish disease diagnoses and treatment monitoring would be much more reliable and preferable, rather than the identification of single biomarkers.

### 3.2 Unique immunological profiles of edge populations indicate the need for developing different immune biosignatures

Special or edge populations usually appear with physiological changes or system immaturities that may produce different immune cell markers (Ciabattini et al., 2018; Abu-Raya et al., 2020; Aulin et al., 2021). Pediatric, pregnant, elderly, and immunocompromised patients are recognized as populations that confound standard TB treatment strategies (Nahid et al., 2016). Determining the TB diagnosis in the pediatric population remains challenging with current routine clinical and laboratory tests (Gunasekera et al., 2022). Both the TST and IFN-γ fail to differentiate diagnoses of TB in pediatric patients. Hence, the WHO have promoted the pediatric TB scoring chart that was invented to help with assessment in limited-resource countries which might be biased (Van Rheenen and Health, 2002). Furthermore, treatment monitoring remains elusive in this population. Most of the time, a clinician only assesses successful treatment based on weight gain and loss of symptoms (Chiang et al., 2020). Although an immune markers-based test promises to solve this issue, no immune-diagnostic or monitoring test is well-developed and validated (Yong et al., 2019; Kumar et al., 2021a). A recent publication found that pediatric TB patients have 20% differentially expressed genes in comparison to adults (Bah et al., 2018). It is very well-known that childhood patients are characterized notably by the significant increase of IFN-regulated genes (Yoshida et al., 2022). Nonetheless, other regulated immune pathway genes may also be upregulated, including the elevation of alternative antimicrobial defense mechanisms (*H2AFJ*, *HISTIH2BG*, and *CTSG*) and multi-functional IL-27 that blocks IL-17 and IRF1 signaling (Bah et al., 2018). In addition to upregulated genes, pediatric TB is also characterized by downregulated genes, with pathways primarily engaged in stimulation of T or B cells (*CD40L*, *CD7*, *ICOS*, *FCER2*, *PTPRCAP*, and *ADAM23*), dendritic cell development (*FLT3LG*), alternative promoters of inflammation (*CD248* and *EDAR*), and inhibition of neutrophil degranulation by *ADORA3*.

Due to their central role in promoting immune responses, monocytes and lymphocytes may reflect a person’s immune status during infection. In TB infection, *Mtb* will target the monocytes for their growth, while lymphocytes are principally responsible for *Mtb* clearance. Monocyte to lymphocyte ratio (ML ratio) has gained interest as a potential biomarker that can classify TB diagnosis even in special populations. A previous report investigating infants born to HIV-positive mothers revealed that the increased ML ratio in peripheral blood was associated with definite TB disease. Although the ML ratio could be a good predictor for TB disease, the ratio showed a modest role in predicting TB disease-free survival (Naranbhai et al., 2014). Another study reported that an extreme deviation of the ML ratio, either high or low (median of ML ratio: 0.36), was correlated with active TB disease. The authors also mentioned that patients with EP-TB and an age of >60 years were more likely to have severe ML ratio alterations (Wang et al., 2015). Since *Mtb* infection can interfere with the development of hematopoietic stem cells (HSCs), the ML ratio might be affected differently among people. For example, *Mtb* hinders HSCs engraftment and uniquely reorganizes HSCs (Khan et al., 2020); *Mtb* infection on HSCs could wreak havoc on the balance of myeloid and lymphoid lineages development in different people (Tamburini et al., 2021). This condition could return to normal with the administration of anti-TB therapy. On the contrary, an earlier study failed to distinguish between LTBI and active TB using the ML ratio (Buttle et al., 2021). Even though the ML ratio showed great sensitivity, the specificity was poor. However, this study discovered a higher ML ratio in males due to less effective immune responses in males. Therefore, the severity of TB disease might be more advanced in male patients. Another study confirmed this finding, showing that the ML ratio (median: 0.5) could distinguish patients with active TB from those of healthy volunteers (La Manna et al., 2017). Despite this, it failed to distinguish active TB from LTBI patients and cured TB patients. Apart from the ML ratio, the neutrophil to lymphocyte ratio (NL ratio) has recently been proposed as a biomarker to distinguish active TB, LTBI, cured TB, and HC. A recent report found that active TB patients appeared to have a high NL ratio compared to both HC and cured TB patients (La Manna et al., 2019). Among those with active TB, a higher neutrophil count and a lower lymphocyte count were identified. Nevertheless, this ratio was gradually decreased by TB treatment, showing the potential of this marker to monitor TB as well. Understanding these potential biomarkers to diagnose TB is considerably important to provide faster and easier confirmation of TB. Therefore, anti-TB drugs can be started immediately. The aforementioned biomarkers were routinely performed in hospital laboratory settings, even in low-resource countries. Although the precision remained inconclusive, these biomarkers may serve as an alternative in limited-resource settings because, as cytokine/chemokine secreting cells, the changes in the ML ratio may contribute to the variation of cytokine/chemokine composition. Further consideration related to other progressive infections possibly affecting the results should be assessed in the implementation of the markers.

Despite substantial differences in immune responses between adults and pediatric patients, some similarities can be observed. There is an increase of lymphocyte and a decrease of macrophage percentages in both adults and children with TB (Herrera et al., 2022). Nonetheless, the downregulation of *IL1RN* and *IL1R2*, which are involved in the generation of the adaptive immune response, is believed to cause disseminated infections in pediatric patients (Bah et al., 2018). Even so, the common immune pathways activated during TB infection are similar between TB adults and children regardless of the different clinical symptoms presented by these two groups (Berry et al., 2010). Several immune chemokines have also been reported to differentiate between active TB and non-TB in children, which showed higher baseline levels of CCL1, CXCL1, and CXCL10 (Kumar et al., 2021a). Those chemokines can decrease with treatment and are useful for treatment monitoring.

In adult TB patients, chemokines have been widely considered as potential diagnostic biomarkers. Indeed, CCL1, CCL3, CXCL1, CXCL10, and CXCL11 were associated with TB disease severity and positively correlated with bacteria load, while CCL1, CXCL9, CXCL10, and CXCL11 could discriminate PTB from LTBI infection in individuals (Kumar et al., 2019b). MCP-1 was also demonstrated to differentiate between PTB, endobronchial TB, and TB pleurisy (Xiong et al., 2016). Baseline levels of plasma CCL2, CCL3, CCL4, CXCL8, CXCL10, CX3CL1, and CXCL1 could predict unfavorable treatment outcomes in PTB (Kumar et al., 2021b). Nevertheless, pregnant women have different immune profiles compared to adults, and transient changes in immunity occur during pregnancy (Abu-Raya et al., 2020). A previous study found that pregnant women with LTBI had lower levels of various proinflammatory cytokines, such as IL-1β, IL-6, and IL-17A, and a higher level of IFN-γ, notably during the second trimester (Naik et al., 2021). These results suggested that latent TB during pregnancy is characterized by a distinct immune profile with high levels of IFN-γ but low levels of other immune markers known to play a role in TB. The mechanisms for this finding were unclear. Previous studies on the mechanisms of TB disease showed that IFN-γ could give negative feedback to IL-1β, IL-6, and IL-17A in some instances (Nandi and Behar, 2011; Eigenbrod et al., 2013), thus high levels of IFN-γ might be associated with a low level of other markers. Additionally, an increase in neutrophil levels was observed during pregnancy and associated with lower IL-6 and IL-17 in TB infection (Sacks et al., 1998; Nishida et al., 2007).

Furthermore, immune markers might be altered by several factors, including age. Studies of immune marker concentration for diagnosis or monitoring TB disease in either elderly or preterm neonates were hard to find. However, physiological changes and the complicated clinical conditions that commonly appear in this population should be noted (Ciabattini et al., 2018; Aulin et al., 2021). TB in the elderly is often associated with the reactivation of quiescent lesions (Caraux-Paz et al., 2021). These activated lesions might be attributable to changes in the immune system related to aging, notably the decline in the ability to reactivate acquired immunity or immune response expressions. Elevated plasma levels of IL-6, IL-1, and TNF-α have been observed in the elderly (Weiskopf et al., 2009; Martín et al., 2017). These markers were previously described as “inflammaging” and are considerable for biomarker-based therapeutic optimization strategies (Franceschi et al., 2018; Aulin et al., 2021). On the other hand, preterm infants have a notable reduction in IL-1β, IL-6, and TNF-α because their innate immune responses are amid the developing process (Delanghe and Speeckaert, 2015). In addition, within several days after birth, preterm and term infants were reported to have higher levels of CRP and PCT, likely related to birth stress (Sharma et al., 2012).

Since TB frequently co-exists in immune-compromised patients, people who live with HIV are the most vulnerable to infection and disease progression risk (Organization, 2015). The increased susceptibility to TB in HIV-infected individuals is likely due to the characteristic immunological effects of HIV that predominantly infect CD4^+^ T cells (Okoye and Picker, 2013; Geremew et al., 2020). Both CD4^+^ and CD8^+^ T cells have been implicated in the immune response against *Mtb* infection (Prezzemolo et al., 2014). However, animal studies have shown that CD4^+^ T cells are more critical than CD8^+^ T cells in the host’s immune response to fight TB (Lin and Flynn, 2015). Additionally, there is growing evidence that TNF-α-dependent macrophage apoptosis is reduced with HIV-TB co-infection (Patel et al., 2007; Walker et al., 2013). These findings indicate that not only is adaptive immune response altered in HIV subjects, but also the innate immune response. A further condition regarding HIV patients that may complicate TB treatment is tuberculosis-immune reconstitution inflammatory syndrome (TB-IRIS). TB-IRIS is an abnormal and excessive immune response against alive or dead *Mtb* that usually occurs in HIV-infected patients (Tadokera et al., 2011; Naidoo et al., 2012). This condition may appear after the initiation of antiretroviral therapy independently of an effective suppression of HIV viremia. TB-IRIS decreases the levels of several circulating inflammatory markers, including IL-6 and Th1/2/17 cytokines (IL-2, IL-3, IL-12, IL-15, and IL-17A) (Ravimohan et al., 2015). The cytokines stimulate T and natural killer (NK) cell responses to activate macrophages and control *Mtb* infection (Gonzalez‐Juarrero et al., 2005; Rausch et al., 2006; Méndez-Samperio, 2010). Therefore, low pre-ART levels of these cytokines in TB-IRIS patients may implicate abnormal innate immune responses and further hamper pathogen clearance. Measurement of immunologic profiles prior to and early after ART initiation for HIV patients is encouraged to predict the treatment response and guide the regimen changes in TB therapy.

Another concern regarding immunocompromised patients is diabetes mellitus (DM). It has been widely reported that DM and TB often appear together, especially in the aging population (Niazi and Kalra, 2012; Lin et al., 2015). DM is known as a risk factor for the conversion of LTBI into active TB (Hensel et al., 2016). The function of neutrophils, macrophages, dendritic cells (DC), NK cells, and other components of innate immunity, as the first-encounter immune responses, are compromised due to metabolic changes in DM patients (Martinez and Kornfeld, 2014; Yew et al., 2017). Hence, type 2 DM patients may be characterized by the decreased secretion of IL-1β, IL-12, IL-18, and IFN-γ, which increases their susceptibility (Stalenhoef et al., 2008). Additionally, DM patients also manifest impaired adaptive immune responses. Previous studies revealed that TB patients with DM had significantly lower frequencies of both myeloid DC and plasmacytoid DC compared with individuals with only TB (Ayelign et al., 2019). Hyperglycemia was reported to cause a lower DC and neutrophils count in TB patients with DM (Kumar Nathella and Babu, 2017; Raposo-Garcia et al., 2017). As an explanation, hyperglycemia may increase adhesion and integrin expression, reduce chemotaxis, defect phagocytes, and reduce microbicidal activity (Raposo-Garcia et al., 2017; Ayelign et al., 2019).

Furthermore, DM also may potentially influence and/or decrease the frequencies of Th1 and Th17 cells due to the increased frequencies of Th2 cells which produce IL-4 (Yamashiro et al., 2005; Kumar Nathella and Babu, 2017). Given all the aforementioned conditions, the incorporation of immunomics into clinical settings would bring more insights to guide safe and effective treatment for these special populations. It is also worth noting that *Mtb*-specific immune responses are probably not homogenous in all populations and might be influenced by the immunity alterations of each specific population. Hence, using generalized potential immune biomarkers to monitor and diagnose TB in these populations may not be appropriate.

## 4 Association of immune profiles to current TB therapies

Interindividual variability (IIV) has been recognized as one of the most important factors causing slow treatment response, treatment failure, and the development of drug resistance in TB treatment (Srivastava et al., 2011). Anti-TB drugs are widely known to exhibit wide IIV on exposure and maximum concentration (C_max_), challenging the finding of optimal dose levels for patients (Zuur et al., 2016; Sturkenboom et al., 2021). IIV affects both the pharmacokinetics (PK) and pharmacodynamics (PD) of the drugs, which is caused by multiple factors, including environment, genetics, demographics, concomitant medications, and comorbidities (Sturkenboom et al., 2021). It is well known that immune recognition, immune response, and immune regulation of *Mtb* determine the occurrence, development, and outcome of the disease. Even though the immune cells’ effects on the PK/PD of the anti-TB drugs has not been fully understood, the general impact of immune cells in altering the drug metabolism enzymes and transporters may be acknowledged (Christensen and Hermann, 2012). Like other infectious diseases, TB may activate the immunological response and release cytokines as a part of its pathophysiology (Dheda et al., 2010). Cytokines are a critical component of the immune response, which act as chemical mediators and bridge the physiological communication among cells throughout the body (Newton and Dixit, 2012). Given the importance of cytokines, understanding how they affect the expression of proteins that influence the outcome of drug therapies is essential. Previous reports demonstrated that cytokines regulate the expression and activity of drug-metabolizing enzymes and transporters, thereby affecting the PK of drugs (Christensen and Hermann, 2012; Sanz Codina and Zeitlinger, 2022). Cytochrome P-450 (CYP) enzymes are involved in the biotransformation of various endogenous as well as exogenous compounds (Tomaszewski et al., 2008). Most anti-TB drugs are metabolized by CYP enzymes (Horita and Doi, 2014). IFN-γ, produced by Th1 in response to TB infections, is known to downregulate the expression of *CYP1A2, CYP2B6, CYP2C8, CYP2C9*, and *CYP3A4* (Wu and Lin et al., 2019). Rifampicin (RIF) is metabolized partially by *CYP3A4*, while *CYP1A2* is needed for hydrazine, one of isoniazid (INH) metabolite, detoxification (Chen et al., 2006; Tostmann et al., 2008). The downregulation expression of these CYPs enzymes causes adverse drug reactions related to isoniazid (INH) and rifampicin (RIF). In addition, IFN-γ, IL-2, and IL-12 are associated with the increased expression of *ABCB1* (encodes P-gp), which is responsible for the low systemic exposure of RIF. The low exposure of RIF as one of key drugs in TB treatment may contribute to the risk of obtaining drug-resistant TB. In addition, the reduced oral clearance of drugs with intermediate to high hepatic extraction ratios, which may affect low binding drugs like INH more, was observed in the patients with elevated proinflammatory cytokines (Mayo et al., 2000; Gausi et al., 2021). A summary of each immune marker and its association with PK parameters are given in **Table 2**.

**Table 2:** Association between immune-marker and pharmacokinetics parameters.

Immune Markers	Pharmacokinetics Alteration	Ref
**IFN-γ**	Decreased expression of *CYP3A4, CYP1A2, CYP2B6, CYP2C8* Decreased volume of distribution Increased expression of *ABCB1, ABCC1, ABCC2*	(Christensen and Hermann, 2012; Young et al., 2020; Sanz Codina and Zeitlinger, 2022)
**TGF-β1**	Decreased expression of *CYP3A4, CYP1A2, CYP2B6, CYP2C9, CYP2C19, CYP2C8*	(Christensen and Hermann, 2012)
**TNF-α**	Decreased expression of *CYP3A4, CYP1A2, CYP2B6, CYP2C9, CYP2C19, CYP2E1, CYP2C8* Decrease volume of distribution	(Christensen and Hermann, 2012; Sanz Codina and Zeitlinger, 2022)
**IL-1β**	Reduce expression of *CYP2C8* and *CYP3A4* Decrease volume of distribution	(Christensen and Hermann, 2012; Sanz Codina and Zeitlinger, 2022)
**IL-2**	Decreased *CYP2B6, CYP3A4* Increased expression of *ABCB1, ABCC1, ABCC2*	(Christensen and Hermann, 2012; Young et al., 2020; Sanz Codina and Zeitlinger, 2022)
**IL-4**	Increased *CYP2B6* and *CYP2E1* Decreased *CYP1A2*	(Christensen and Hermann, 2012; Young et al., 2020)
**IL-6**	Reduce expression of *CYP2C8, CYP2C9, CYP2C19, CYP3A4, CYP2E1* Decrease volume of distribution	(Christensen and Hermann, 2012; Sanz Codina and Zeitlinger, 2022)
**IL-10**	Increased expression of *CYP3A4* Decrease volume of distribution	(Christensen and Hermann, 2012; Sanz Codina and Zeitlinger, 2022)
**IL-12**	Increased expression of *ABCB1, ABCC1, ABCC2*	(Young et al., 2020)

However, it is worth mentioning that not only do immune profiles affect the PK of anti-TB drugs, but anti-TB drugs can also directly or indirectly regulate immune profiles (Park et al., 2021). Anti-TB drugs alter the immune system by indirectly affecting microbiota composition or directly affecting immune cell functions (Ubeda and Pamer, 2012; Moffatt et al., 2019). Thereby, the clinical outcomes and treatment response vary between patients. For instance, INH induces the apoptosis of activated CD4^+^ T cells and impairs the production of *Mtb*-specific IFN-γ and anti-CFP-10 antibody in latent TB patients (Tousif et al., 2014). Furthermore, the administration of RIF disturbs the cytokine cascade by suppressing IL-1β and TNF-α, and inducing IL-6 and IL-10 secretion (Ziglam et al., 2004). Other anti-TB drugs also are known to modulate immune profiles and are further summarized in **Table 3**. Considering the previous findings for both immune profiles and anti-TB drug association, comprehensive evaluation and understanding of TB pathophysiology, immune pathways, and PK profiles of anti-TB drugs should be carried out during biomarker validation for personalized medicine services in TB treatment.

**Table 3:** Anti-TB drugs modulation to immune profile" into "Anti-tuberculosis drugs modulation to immune profile.

Anti-TB drugs	Altered Immune cells expression	Ref
**INH**	Induces apoptosis of CD4^+^ T cells in *Mtb*-infected. Impairs the production of *Mtb*-specific IFN-γ and anti-CFP-10 antibody.	(Tousif et al., 2014)
**RIF**	Suppresses the formation of T cells, phagocytosis by macrophages, and expression of TNF-α (at high dose). Inhibits the secretion of IL-1β and TNF-α. Increases the secretion of IL-6 and IL-10. Suppresses LPS-induced production of iNOS, cyclooxygenase-2, IL-1β, TNF-α, and prostaglandin E2 in microglial cells.	(Nilsson, 1971; Gupta et al., 1975; Mlambo and Sigola, 2003; Wilson and Jordan, 2004)
**PZA**	Decreases the secretion of proinflammatory cytokines and chemokines, such as IL-1β, IL-6, TNF-α, and MCP-1. Increases the expression of adenylate cyclase and peroxisome-proliferator activated receptors.	(Manca et al., 2013)
**BDQ**	Promotes the formation of lysosomes, phagocytic vesicle membrane, vacuolar lumen, hydrolase activity, and lipid homeostasis in both naïve and *Mtb* infected macrophages. Suppresses basal glycolysis. Reduces glycolytic capacity in macrophages. Initiates phagosome–lysosome fusion and autophagy.	(Giraud-Gatineau et al., 2020)
**CFZ**	Inhibits TLR2-and TLR4-mediated NF-kβ activation and TNF-α production. Induces apoptosis of macrophages. Increases caspase-3 activity in macrophages.	(Fukutomi et al., 2011; Urbanova et al., 2016)

CFZ, Clofazimine; BDQ, Bedaquiline; INH, Isoniazid; PZA, Pyrazinamide; RIF, Rifampicin.

### 4.1 Biomarker-guided approach for personalized medicine of anti-TB drugs: a potential application

To overcome IIV, TDM has been utilized to adjust the dose following patient’s PK/PD profile. This can reach well-defined PK/PD targets associated with the efficacy and toxicity of drugs (Alsultan and Peloquin, 2014; Lange et al., 2020). Most of the dose adjustments that have been done in clinical settings follow the PK/PD indices of specific anti-TB drugs (Alsultan and Peloquin, 2014; Sturkenboom et al., 2021). Nonetheless, substantially unpredictable variations in treatment response remain for some patients. Demographic characteristics, clinical conditions, and genetic variants of metabolic enzymes or transporters of anti-TB drugs are commonly identified as the source of IIV affecting the treatment response of patients. Apart from the previous findings, other sources of IIV, in relation to pathogen characteristics, a patient’s immune response, or the effects of inflammation on PK, should also be explored (Young et al., 2020; Aulin et al., 2021). In the near future, precision medicine for TB should not only depend on the pharmacological aspects of anti-TB drugs, but rather also focus on biomarker-based treatment decisions and monitoring (Alsultan and Peloquin, 2014; Aulin et al., 2021). Biomarkers hold the potential to inform treatment strategies and aid decisions throughout all phases of infection (Delhalle et al., 2018). However, the current application of biomarkers in TB treatment has primarily been introduced for diagnosis.

Recently, immune profile biomarkers have been of interest to quantify treatment efficacy in relation to treatment response due to the length of time needed for bacterial culture and the difficulty of sputum production (Thorsted et al., 2020; Mensah et al., 2021). During TB infection, the immune response is triggered by *Mtb*, producing several immune biomarkers. To guide and optimize TB treatment, immune biomarkers should ideally reflect the underlying pathogen load. In addition to the role of immune biomarkers for treatment monitoring and outcome prediction, the effect of immune biomarkers on IIV of anti-TB drugs PK also should be evaluated. It is widely known that infection induces inflammation effects on the absorption, distribution, metabolism, and elimination processes of drugs (Radke et al., 2017; Charlton and Thompson, 2019). Hence, immune biomarkers that capture infection-induced inflammation effects and predict IIV on anti-TB drug PK parameters are significantly relevant for further individualizing TB treatments.

### 4.2 The current state of biomarker development and technical considerations for immunoassay in TB

Early and accurate detection of active TB is crucial to prevent transmission of TB, yet existing diagnostic procedures are insufficient. Despite the development of several immunoassays for determining antibodies or antigens in serum samples, only a few diagnostic methods are available (Kashyap et al., 2007). Typically, ELISA is the primary analytical tool to quantify *mycobacterium*-specific components (Kashyap et al., 2007; Reither et al., 2009), host antibody response (Ben Selma et al., 2010; Zhu et al., 2012), and cytokine/chemokine production (Hur et al., 2013; Indrati et al., 2022) for diagnosis and monitoring treatment (Kim et al., 2021). Kashyap et al. employed an indirect ELISA method to diagnose TB by monoclonal antibodies against Ag 85 complex, which yielded high sensitivity and specificity of up to 82% and 86%, respectively. Recently, a quantitative lateral flow assay was established for adjunct diagnostics and treatment monitoring of TB and COVID-19 (Pierneef et al., 2022). Analysis of seven host sera proteins exhibited potential in differentiation of LTBI and TB, HC and COVID-19 patients, and severe COVID-19 and TB (Pierneef et al., 2022). Additionally, there are several alternative approaches to detect TB in serum and urine, including dot-immunobinding assay (Bentley-Hibbert et al., 1999), nucleic acid amplification (D’Amato et al., 1995), the application of two-dimensional polyacrylamide gel electrophoresis (2DGE), and liquid chromatography-tandem mass spectrometry (LC-MS) (Kashyap et al., 2005).

Flow cytometry is an alternative approach frequently used to evaluate various cell surface markers and intracellular cytokine production, permitting the characterization of distinct immune cell types. Several analyses using this method manifest the possibility of distinguishing between active, LTBI, and uninfected patients (Harari et al., 2011; Pollock et al., 2013; Estévez et al., 2020a), and assessing treatment efficacy (Ahmed et al., 2018) as well as drug susceptibility (Hendon-Dunn et al., 2016).

**Figure 2:**
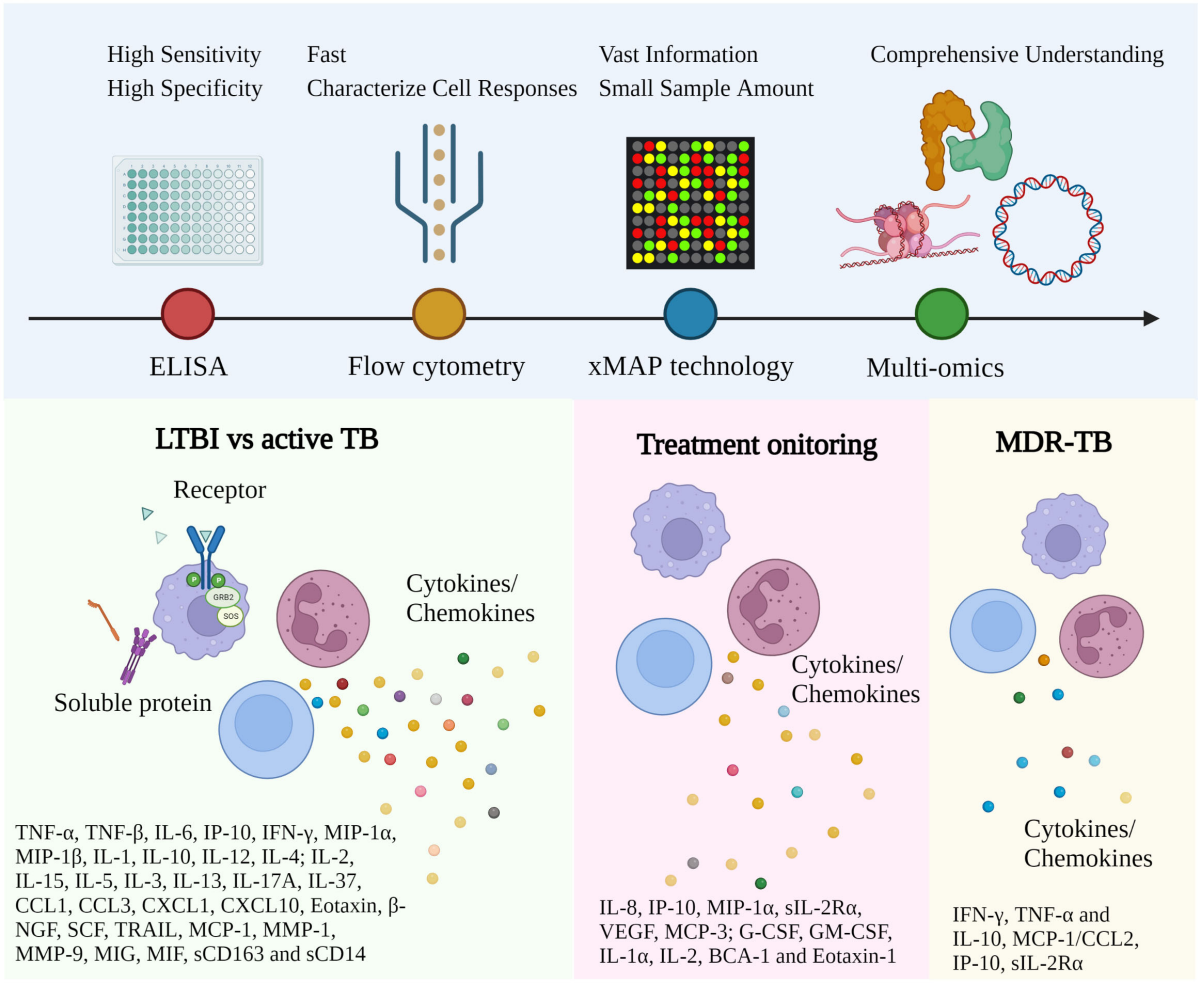
Representative immune biomarkers corresponding to TB diagnosis and management. The approach utilized for TB investigation has been gradually converted from conventional ELISA to high-throughput technology with multiple advantages. More and more immune biomarkers have been reported for different disease stages and types. Robust and affordable high-throughput technologies and data analysis facilitate the discovery of clinically relevant biomarkers *via* well-established study designs. (ELISA, Enzyme-linked immunoassay; xMAP, Multi-Analyte Profiling; LTBI, Latent TB infection; MDR-TB, Multidrug-Resistant Tuberculosis).

More recently, multiplex bead array assays have emerged in analytes quantification (Elshal and McCoy, 2006) simultaneously due to their capability to provide a vast quantity of bio-information from a small sample size for a better prognosis, diagnosis, and treatment (Ahsan, 2021). The multiplex immunoassays make use of conventional immunoassay methods that capture circulating antibodies or proteins by using either antibodies or peptides/proteins as binder molecules (Ahsan, 2021). In suspension experiments, the targeted ligands are immobilized on color- or size-coded plastic microspheres, and flow cytometry is used to identify particular fluorescent signals (Ellington et al., 2010). Bead-based flow cytometry technologies that enable multianalyte measurements in clinical diagnostics are already widely accessible on commercial platforms (Spindel and Sapsford, 2014). By using multiplex methods, such as xMAP technology developed by Luminex or Bioplex, immunomics is by far strengthened in reducing technical burden. The Luminex xMAP system is one of the pioneers (Luminex Corp., USA). It has been employed in numerous recent studies to investigate and validate immune biomarkers for TB diagnosis (Phalane et al., 2013; Xiong et al., 2016; Kumar et al., 2021a; Mutavhatsindi et al., 2021), treatment monitoring (Kumar et al., 2020), and recurrence prediction (Sivro et al., 2017). Conversely, a conventional ELISA experiment is laborious and usually takes days to quantify multiple immune molecules. However, a multiplex system facilitates simultaneous evaluation and demonstrates correlation in data with ELISA within several hours (Wang et al., 2005). On another scale, Meso Scale Discovery (MSD), a global multiplex analysis to characterize innovative assays for biological molecules, is also worth considering. Sharing comparable specifications, both Luminex and MSD are applicable for multiple immune profile analyses (Chowdhury et al., 2009). However, while MSD has a wider dynamic range and sensitivity, Luminex’s strength is emphasized in its precision (Sourial et al., 2009; Günther et al., 2020), which may be more suitable for the considerable input of biomarkers across the wide range of personalized treatments. Therefore, despite the need for further validation, multiplex technology, especially Luminex, is taking steps to unlock complex cytokine networks and reveal new biosignatures for TB. The remarkable milestones of these TB-related immunological investigations are briefly depicted in **Figure 2**.

## 5 High-throughput immune biomarker quantification: A new member of the omics family

The immune system is a complicated and pervasive network that contains adaptive responses of numerous cell types and intracellular signals to recognize pathogens or stimuli and cross-communicate to perform protective functions (2013). With respect to this complexity and universality, there is no single golden standard for what to invest in among multi-omics layers. Whilst a single omics layer can only provide insight into a particular aspect, immunological studies tend to adopt different omics measurements to build rigorous profiles of samples at diverse levels, including genetics, epigenetics, transcriptomics, proteomics, metabolomics, and cellomics (Yu et al., 2019; Ota and Fuijo, 2021). In this part, we briefly discuss different technologies that can be applied in immunomics.

Genomics characterizes genetic diversity from single-nucleotide polymorphisms (SNPs) and insertion/deletion (InDels) to large-scale mutations that trigger polymorphism in the individual immune system (Netea et al., 2016). Popular techniques include PCR-based methods such as mixed-linker PCR (ML-PCR), fast ligation-mediated PCR (FliP), ligation-mediated PCR (LM-PCR), and DNA sequencing such as Next generation sequencing (NGS) and microarray.

Uninvolved in the changes in the nucleotide sequences, epigenomics evaluates chromosome accessibility, structure, and any modification of chromatin, DNA, or histone such as methylation and acetylation. The most commonly used techniques to capture chromatin phenomena are assayed for Transposase-Accessible Chromatin using sequencing (ATAC-seq), DNase-seq, and FAIRE-seq. By using the high throughput sequencing technique, both genomics and epigenomics facilitate huge reference genome databases for shotgun analysis with a static link to the organism. However, besides the need for high-performance machine installation, genome reconstruction by bioinformatic software also requires advanced training and troubleshooting, disregarding the fact that many sequenced elements’ activity cannot be fully identified (Chu et al., 2021; Ahamad et al., 2022).

Transcriptomics depicts RNA profiles under coding and non-coding transcripts of either a single cell or a bulk level. Unlike traditional PCR, which only amplifies a limited number of genes simultaneously, currently used transcriptomic techniques such as RNA-sequencing (RNA-seq) and microarray can quantify the entire transcriptome or the large-scale targeted analysis of most well-known genes. However, aside from handling errors during the long process of RNA extraction and sequencing, there is an undeniable disadvantage: RNA polymorphism is not always reflected in protein translation and functional expression (Chu et al., 2021; Ahamad et al., 2022).

While the genome is generally constant, proteome differs within cells and with time. Therefore, the large-scale study of proteins, proteomics, is more predictably complicated than other omics studies. In the immune system, immune molecules are often detected by either traditional immunofluorescent staining, 2DGE, or modern immunoassays such as ELISA, EMIT (enzyme multiplied immunoassay technique), and mass spectrometry-based approaches: LC-MS and MALDI-TOF (Matrix-Assisted Laser Desorption/Ionization-Time of Flight). Proteomics describes a direct link to organism phenotypes by surpassing the genomics-based approach regarding the correlation to functional expression. Furthermore, as the direct readout of the phenotype, metabolomics is suitable for quantifying metabolic responses to pathophysiological stimuli or genetic modification of a living organism. Metabolomics and its sub-field, lipidomics, can deal with complex metabolites at a high resolution to provide a reliable link between phenotypic characteristics and metabolic profiles. This technology is also helpful for discovering metabolism-centric biomarkers for TB (Long et al., 2022). However, the variation of metabolites is unavoidable, which may lead to several sampling artifacts and fluctuations (Chu et al., 2021; Ahamad et al., 2022). Additionally, metabolite and protein annotation is not always robust, the quantitative information is often limited, quality assurance/quality control (QA/QC) is troublesome, the cost is high, and the technical requirements are affordable only for specialized laboratories.

It has been highlighted that a single omics approach does not decently capture the whole biological process of TB and is unable to realize the true potential of omics for clinical research and implementation (Subramanian et al., 2020). The combination of transcriptomics and metabolomics to discriminate TB progressors improved the sensitivity of the test by up to 12% while still preserving the specificity value of 80% (Duffy et al., 2019) in comparison to using transcriptomic signatures alone. The upregulation levels of metabolite cortisol and the genes *SOC1* and *DDIT4* also were found to be associated with the immuno-metabolic pathways of TB progression.

**Figure 3:**
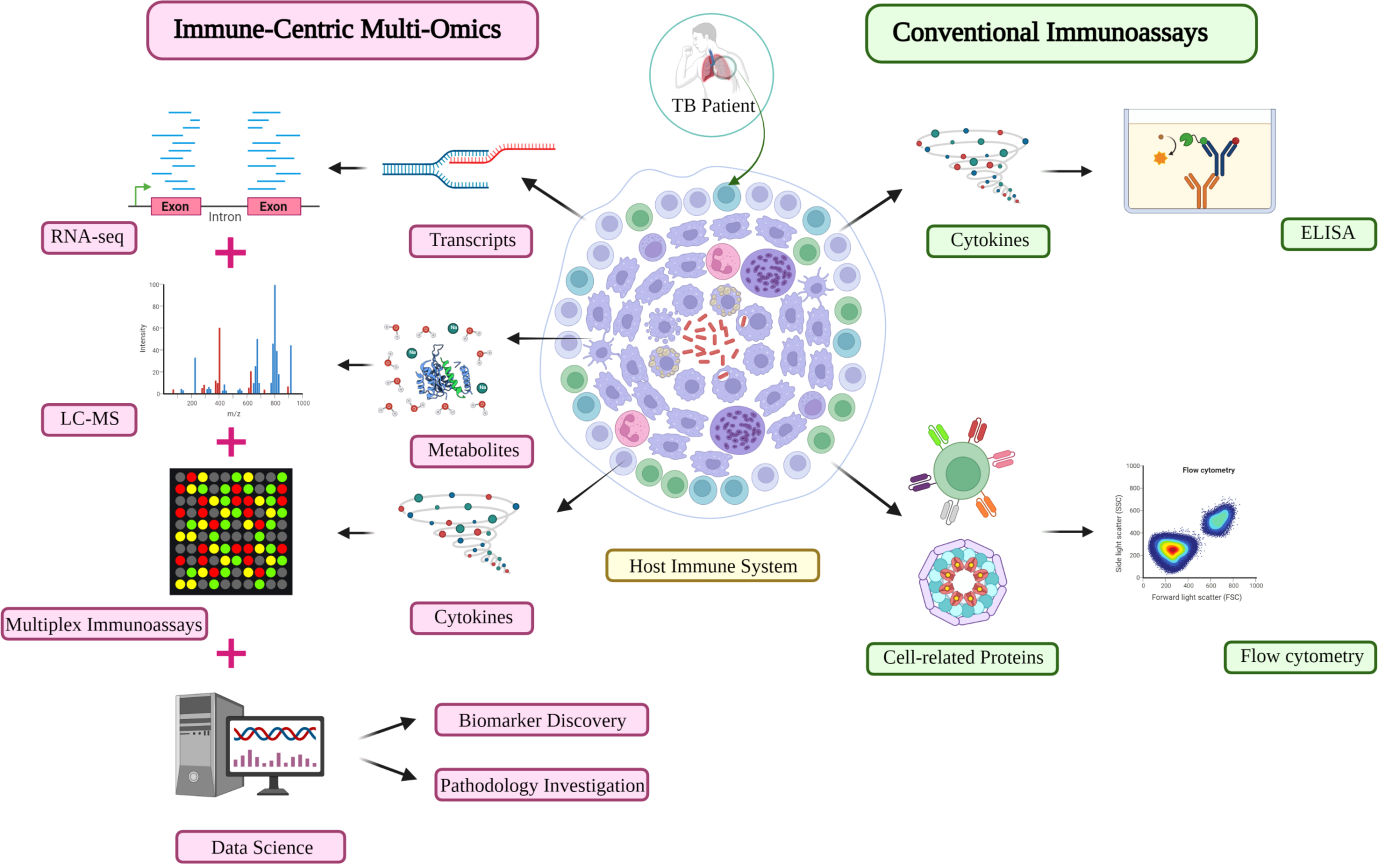
An immune-centric approach for TB investigation in the omics era. Comparison of the high-throughput approach (left) and conventional assay (right) for immune profiling investigation in TB diagnosis and monitoring. Other matured omics technologies are capable of providing complementary information about the immune profiles of TB patients. LC-MS, liquid chromatography-mass spectrometry; TB, Tuberculosis.

Considering the drawbacks of previous omics studies and the need for direct references for TB clinical practice, immune profiling emerges as a promising solution that directly measures the immune response to pathogens, including all immune-related molecules and their regulators or targets. High-throughput technology, e.g., xMAP, is a game changer for immunologists (**Figure 3**). Immune molecule quantitative analysis not only ignores the transient nature of metabolites and the inconstant reflection from genes to proteins, but can also measure immune functioning during the development of therapies. It easily limits the waiting time for the clinical manifestations of toxicity, and overcomes the robustness problem of the former proteomics by integrating multiplex technology. Therefore, it increases the detectability and decreases the risk of unexpected metabolites and the number of subjects required in a trial.

However, immunomics creates a complicated scenario because it studies and manipulates the genes and proteins involved in immunologic functioning and the complex cellular microenvironment. The future of immunomics lies in the continued pursuit of validation of immunologic markers correlated with clinical outcomes, which is now limited, and many technologies are still in their infancy (Tremoulet and Albani, 2005; Chu et al., 2021; Ahamad et al., 2022). In other words, this is a chance to build up a platform worthy of investment, which only requires intense studies and improvement but has several advantages the previous one lack.

## 6 Discussion

**Figure 4:**
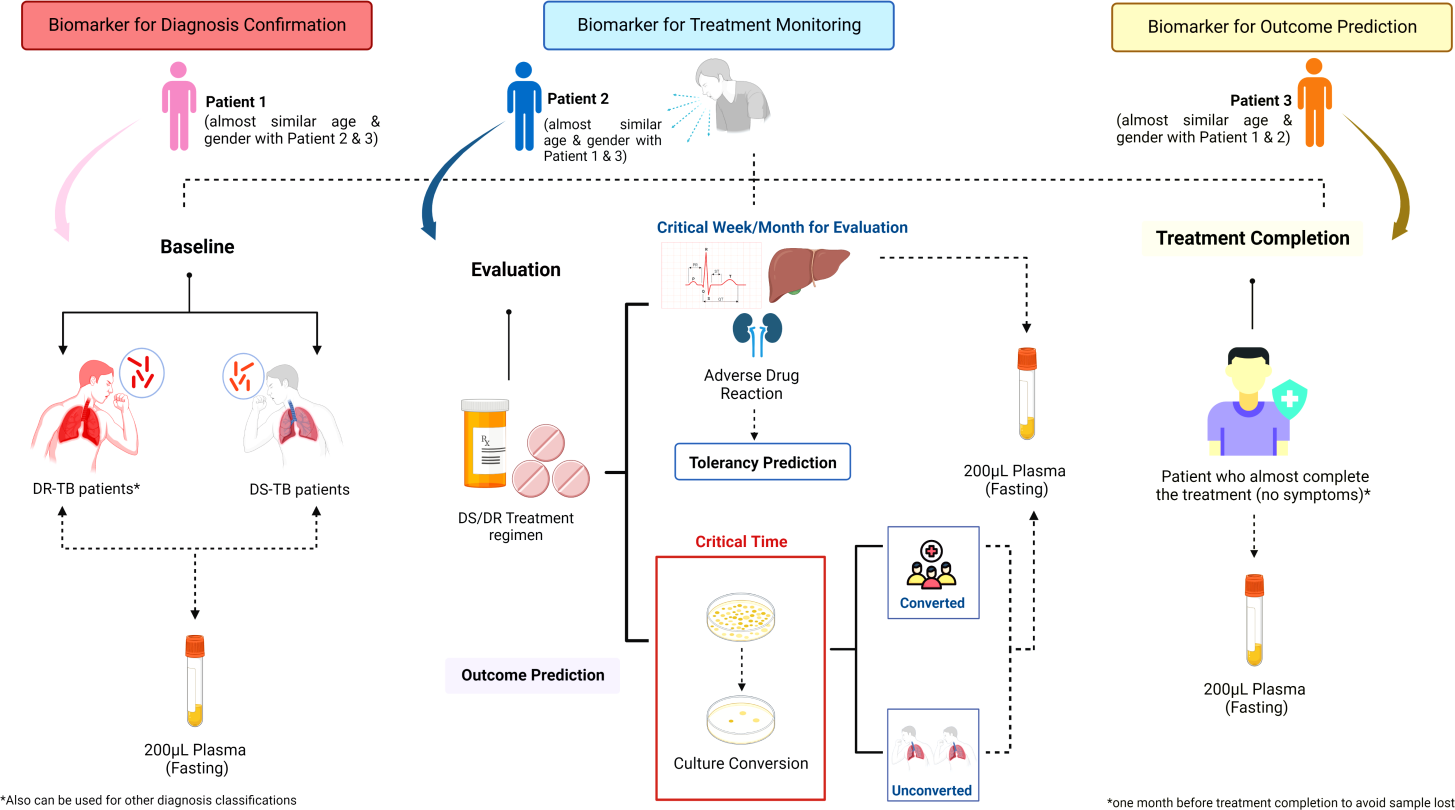
Sample collection strategy of immunological biosignature discovery. The sample collection for immunological biosignature discovery should focus on three critical phases of tuberculosis (TB) treatment: 1) Baseline; 2) Evaluation; 3) Treatment completion. The samples taken in the baseline before TB treatment is started could be used as biomarker for diagnosis confirmation. Meanwhile, the samples collected in the evaluation phase could be used to identify biomarkers for treatment monitoring. Further, the biomarkers could predict the early bacterial conversion and/or adverse drug reaction potency. The samples taken during the treatment completion could be helpful to predict the successfulness of treatment and possibility of future recurrence. Contributions to each phase might be from either the same patients or different patients of a similar age and gender compared to the patients enrolled in other phases. DS, Drug Susceptibility; DR, Drug Resistance; TB, tuberculosis.

The concept of personalized medicine highlights the importance of a patient-tailored regimen by considering individual physiological and pathological factors. As has been stated earlier, one of the cores of personalized medicine is the translational process from the bench to bedside and vice versa (Hartl et al., 2021). As multi-omics appears to be one of the key components of translational personalized medicine, integrating multi-omics from early development (translational medicine) into late development (personalized medicine) is of the utmost importance (Shakhnovich, 2018; Hartl et al., 2021). Each omics discipline has different values for different clinical settings. Considering the nature of this infectious disease, which needs the optimal treatment at the earliest timing, immune profiling may be a game-changer in personalized medicine for TB (Di Martino et al., 2020; Wicha et al., 2021). The current approach to confirming TB diagnosis needs drug-susceptibility testing in the form of bacterial culture, and it has a long turnover time for results (Organization, 2020). The validated molecular biomarkers, especially the immune profile or a panel containing heterogeneous biomarkers, could be used to classify differential diagnoses of TB infection from the beginning of treatment, eventually saving much treatment cost and time (Yong et al., 2019). Furthermore, the markers can be utilized in treatment monitoring and predicting which patients have a higher risk of treatment failure (Duffy et al., 2019; Yong et al., 2019). Regimen changes and dose optimization could be done sooner rather than waiting for the radiological changes, acid-free bacilli staining, culture conversion, and evaluation. A strong point of immunomics is its small sample volume with no time-sensitive sampling needed. Thus, this approach is more convenient for patients and real clinical-setting practice. We have suggested a framework for facilitating the research and development of immune biomarkers (**Figure 4**). It is comparable with the study design and technical requirements of other functional omics platforms, such as proteomics, metabolomics, and lipidomics. Indeed, rapid diagnosis and excellence treatment monitoring are the keys to eradicating TB (Alffenaar et al., 2020). Previously, the WHO has endorsed a new target product profile with regards to a non-sputum-based test capable of detecting all forms of TB by identifying characteristic biomarkers or biosignatures (Organization, 2014). The POC biomarker test is intended to enable the diagnosis of PTB and EP-TB, as well as TB in pediatric.

The COVID-19 pandemic has jeopardized TB treatment and further substantially changed the conventional research and development (R&D) approach in many aspects (Acosta et al., 2022; Petersen et al., 2022). Virtual utilization of advanced technology, data-based, data-sharing, and collaborative research are now preferable (Hartl et al., 2021; Acosta et al., 2022). The further integration of multi-omics and put artificial intelligence (AI)-based analysis have also shown great potential for digital biomarker endpoints and diagnoses. Considering all the opportunities to improve TB treatment strategies that have arisen during the COVID-19 era, several pitfalls challenge the success of the new current R&D approach (Acosta et al., 2022). It should be noted that biomedical data frequently have high numbers of missing data or clinical information to validate the findings. It is sometimes more appropriate to fill these gaps through statistical procedures, such as multiple imputations, even though, in some circumstances, it is possible to simply exclude patients with missing data prior to training. Nonetheless, this approach might further introduce bias into the analysis. Although large datasets are needed to train adequate model predictors, a data set with various covariates or features would result in better predictions, due to a more comprehensive evaluation of variable combinations. Additionally, longitudinal or repeated-sampling study designs also have certain advantages, such as increased statistical power and better PK-/PD-profile modeling. It should be noted that extensive data collection would be challenging at times and need multi-nation collaboration. Another foremost challenge to address is the geographical barrier in data collection, which limits the evaluation of potential interethnic diversity, particularly in genetics, gene expression, and PK-/PD-related profiles to anti-TB drugs (Olafuyi et al., 2021). Moreover, discovered biomarkers should also be validated with large populations. In a recent metabolomics study from Indonesia, TB-DM patients were further characterized by lower levels of glycine, serine, threonine, and homoserine compared to TB patients and HC (Vrieling et al., 2019). This result was consistent with their previous publication focused on other ethnicities. Thus, these markers could be considered as potential POC biomarkers tests to classify TB-DM patients. Just as importantly, the assimilation and collection of multi-modal data, including multi-omics, from collaborative research also needs the assurance of data safety. Hence, trusted central centers should be established to train the models, build safe data storage, ensure data standardization, and finally distribute the models to collaborators for knowledge transfer and data sharing.

Our recent review paper has proposed a novel strategy by incorporating semi-automated TDM using MIPD as a pillar of TB treatment (Jayanti et al., 2022). MIPD-based TDM has three approaches for its implementation, which incorporate a mechanistic approach, physiologically based pharmacokinetic modeling and simulation (PBPK), a data-driven approach (population PK), and a combination of both. Thus far, the population PK model is the most common tool used in MIPD-based TDM to identify the source of IIV in PK parameters through the exploration of different available covariates (Polasek et al., 2019). These models are helpful in quantifying the characteristics of biomarker emergence and decline, their connection with PK or drug exposure, and the measurement of specific sources of IIV and patient-associated predictors (Vinnard et al., 2017; Aulin et al., 2021). Another important potential use of pharmacometrics models for biomarkers is clinical outcome relationships, which may use the biomarker results as the PD target (Thorsted et al., 2020; Aulin et al., 2021). Since the MIPD-based TDM models may facilitate mechanism-based integration, model-based approaches play a pivotal role in reaching this aim.

**Figure 5:**
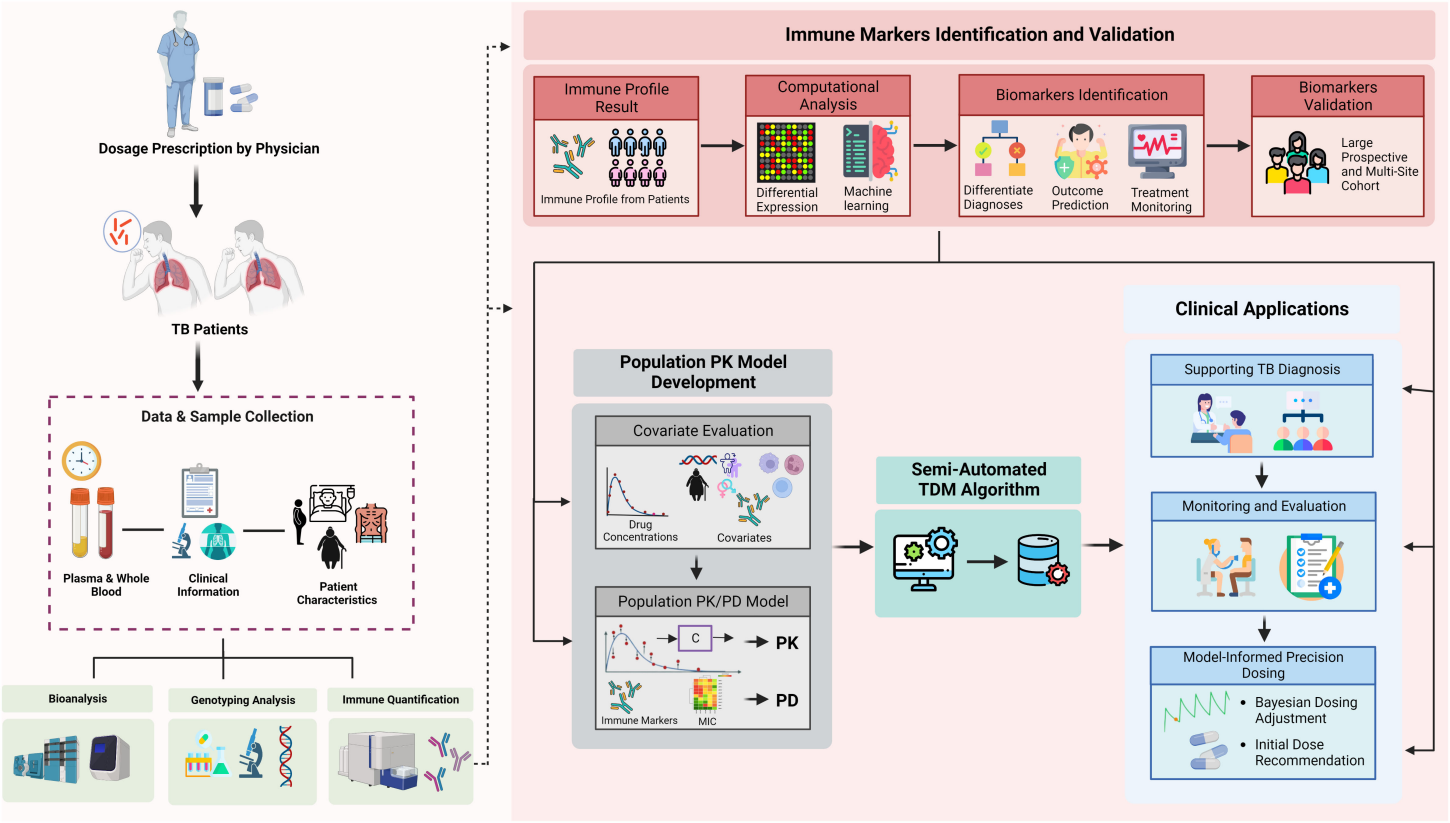
Integration of immunomics biosignature into semi-automated therapeutic drug monitoring application. Immune profile quantification in relation to a patient’s outcome and medical records are used to: (1) validate representative markers for TB diagnosis, outcome prediction, or susceptibility; (2) differentiate diagnosis of TB patients and outcome predictions; (3) provide initial optimal dose; (4) provide additional solid input for treatment monitoring; and (5) semi-automated TDM under population PK model evaluation. The quantified immune profile from each patient will be taken as part of comprehensive covariates evaluation during population PK model development. Our current semi-automated TDM algorithm takes PK results into account for each specific drug, immune markers (as both covariates and PD marker), ethnic differences, and clinical symptoms to provide optimal dose suggestions. Furthermore, the result of immune markers also can be provided in the TDM report to support the monitoring and evaluation phases. TDM, therapeutic drug monitoring; TB, tuberculosis; PK, pharmacokinetics; PD, pharmacodynamics; CL, clearance; Vd, volume of distribution; Ka, absorption rate; Tv, typical value.

We have developed an informatics framework for the MIPD-based TDM algorithm. The algorithm allows us to monitor anti-TB drug concentrations in patients and suggests the optimal dose initiation and adjustment following the model-based estimation PK profile. Established collaboration with other high-burden TB countries allows the transfer of knowledge and technology to attain TB eradication through personalized medicine implementation as the end goal of the partnership. Our current models have been developed explicitly following specific ethnicities (Cho et al., 2021; Soedarsono et al., 2022), to ensure the PK estimation and significant covariates found are more precise and accurate. Considering only PK for personalized medicine might not be appropriate. As is widely known, PD parameters are essential to provide precise dose predictions (Thorsted et al., 2020). Nonetheless, this raises concern as the majority of patients would most likely produce no sputum before or after treatment. A previous study also found that the culture and smear test as a prognostic treatment outcome evaluation showed poor performance (Horne et al., 2010). Hence, it is about time to find the surrogate markers that can indicate early drug efficacy and accurately predict relapse and/or treatment failure which would significantly reduce the cost of TB treatment. Furthermore, even though the non-linear mixed-effect model could handle the sparse sampling approach at random-post dose points, samples taken near C_max_ or distribution time of the drugs would be preferable (Free et al., 2011). Our current proposed strategy is to incorporate the data reflecting the immune profiles of TB patients into our well-developed algorithm of MIPD-based TDM, which is further illustrated in **Figure 5**. In order to achieve the aforementioned aims for this approach, a small sample size will not be sufficient. Considering the dynamics and kinetics changes of biomarkers throughout the infection and treatment phases, the novel discovered immune and other molecular biomarkers should be further validated in a large-scale cohort with the prospective observational design (Aulin et al., 2021; Bonaguro et al., 2022; Sanz Codina and Zeitlinger, 2022).

In conclusion, TB management is not a new issue, but it could take a long time to accomplish the End TB Strategy’s mission, especially in developing countries and due to the pandemic. COVID-19 has suspended the global progress of TB control, highlighting the lack of accessibility and adaptability of current TB diagnosis and therapies. Immune profiling is emphasized in this paper as both a self-sufficient and an additional instrument to improve the existing TB control strategies in an unbiased manner. Our reviews summarized the relevance of immune biomarkers in TB management and characterized the integration of immunomics into the current TB roadmap of research and development. High-throughput immune profiling benefits not only the validation of new immune biomarkers for TB diagnosis, but also the outcome prediction, treatment monitoring, and anti-TB drug dose optimization, which will speed up TB control globally, and ensure “the world without TB” will not be a far off future.

## Author contributions

Conceptualization, Y-SC, NL, and J-GS; Writing—Original Draft Preparation, VT, LD, RJ, HT, TH, NL, and J-GS; Writing—Review & Editing, VT, LD, RJ, HT, NL, Y-SC, and J-GS; Visualization, VT, LD, and RJ; Project Administration, NL; Supervision, Y-SC and J-GS; Funding Acquisition, J-GS. All authors have read and agreed to the published version of the manuscript.
